# Parasites alter interaction patterns in fish social networks

**DOI:** 10.1098/rspb.2025.0793

**Published:** 2025-05-28

**Authors:** Michael Reynolds, Fredric Windsor, Sarah Perkins, Joanne Cable

**Affiliations:** ^1^ School of Biosciences, Cardiff University, Cardiff CF10 3AX, UK

**Keywords:** host–parasite networks, ectoparasites, *Gyrodactylus turnbulli*, *Poecilia reticulata*, network dynamics

## Abstract

Social networks influence the spread of parasites through populations. Although we know how parasites are transmitted as a product of social interactions, we have a limited understanding of how social networks are affected by parasites over time. Host–parasite interactions and the networks they form, are typically examined as static networks, and while topological descriptions at a specific time point are useful, both behaviour and the infection process are dynamic. By monitoring replicate populations of Trinidadian guppies (*Poecilia reticulata*) daily before and during infection with the ectoparasite *Gyrodactylus turnbulli*, we show how parasitism drives social network dynamics. Specifically, infected individuals increased their connections in networks affected by parasitism. In contrast, uninfected control shoals showed no change in network metrics. The structure of subnetworks (motifs) and networks, however, did not change in response to infection status. These findings provide further evidence of reciprocal host behaviour–parasite feedback mechanisms, and highlight that infected fish alter their interactions in order to ‘off-load’ their parasites. Understanding how these reciprocal interactions affect the structure and function of natural systems, as well as understanding how these interactions may alter with future environmental change, are key areas of future research.

## 1. Introduction

Complex social networks are observed across a range of different organisms, from fish [1] to humans [2]. Interactions within these networks can take many forms, from mutualistic through to antagonistic [3]; however, a commonality across social interactions, is that they typically involve close contact between individuals. As such, the structure of social networks, as well as the identity and strength of the interactions therein, have important implications for population-level processes, such as disease transmission [4].

The structure of social networks is influenced by both biotic and abiotic factors [5]. Firstly, the behavioural ecology of the organism in question has a large bearing on the structure of the social network [6]; at the extremes, social organisms form tightly interconnected networks, with many connections between individuals, whereas more solitary organisms may have far sparser social networks [7]. Secondly, environmental conditions alter social networks through their influence on the habitable area (e.g. restricting available habitat [8]); as well as the interactions on the behaviour of the individuals themselves (e.g. temperature and activity rates [9]). As well as these direct drivers of social networks, there are reciprocal feedback and indirect influences. The transmission of parasites, for example, can be influenced both by social interactions, their hierarchical structure and the social networks they form [10].

Although many studies have investigated social network structures, and the implications at individual, population and community levels, most assessments are static (i.e. focusing on a single point in time) or use a dynamic approach, but with simulated networks [11]. Existing studies that have investigated dynamic responses of social networks have typically focused on the removal or replacement of key individuals [12,13]. These studies have shown that social networks can respond at a series of different scales, from changes in individual behaviour to shifts in the topology of the overall social network. There remains limited information on how networks respond to other types of perturbation, e.g. environmental change or parasitism, despite there being a wealth of information on individual behaviours [14].

Parasites are central in all ecological systems [15] and present a potentially significant disruptor of social networks [16]. Interactions between hosts and parasites can generate a range of changes from individuals through to entire networks [17]. At the individual scale, parasites can affect all organism-level traits from physiology to fecundity [18]. In fish, infected individuals showing increased sociality [19] may try off-loading parasites onto uninfected or less infected individuals [20]. This process of ‘off-loading’ has been observed in other experiments focusing on dyads (see [20]), and appears to be an individual-level response aimed at reducing the negative effects associated with high parasite burdens and diluting their parasites amongst potential hosts (see [21]). Indeed, previous studies have shown that a higher contact rate between individuals enhances the transmission of parasites [22] and could thus be used to reduce individual burdens. Furthermore, greater shoal sizes, and therefore higher dilution, have been shown to act as an anti-ectoparasite mechanism in other shoaling fish species [23]. Individuals may also exhibit behavioural traits that select for a reduction in parasitism, e.g. seeking water conditions that are less favourable for the parasites [24]. Other responses, such as avoidance, have been shown in host–parasite systems; however, these processes take longer to emerge (multiple weeks) and only appear to occur at very high levels of infection [25]. Across subnetworks, also known as motifs [26], interactions between individuals can be altered by parasites, although motifs are an underutilized tool [27]. Three-mode motifs, or ‘triangles’, can provide additional information, including indirect interactions, and thus are an intermediate structural unit between individuals and networks [26]. In the case of social networks and parasite transmission, motifs can provide information on intermediate hosts, as well as transmission pathways within subnetworks of the wider social network. For example, they can be used to identify when an uninfected individual is in contact with multiple infected individuals or *vice versa*. Finally, entire social networks may become more or less connected in response to parasites, depending on the mechanisms through which parasites affect individual and group behaviours [28].

Here, we investigated how social networks in populations of the Trinidadian guppy (*Poecilia reticulata* Peters 1859) respond to parasitism by *Gyrodactylus turnbulli* (Harris 1986) over time. Through a series of controlled experiments, we aimed to understand how infection of individual *P. reticulata* with *G. turnbulli*, and subsequent transmission, affected the host social interaction networks. We hypothesized that social networks would respond to parasitism at individual, motif and network scales in the following ways:

(1) Social interactions among individuals will change after infection, based on two previously observed mechanisms: (i) out-degree of infected individuals will increase (shedding or off-loading) and (ii) in-degree of uninfected individuals will increase (acquiring);
(2) Motifs associated with the transmission of parasites will increase in frequency as infection increases in prevalence and intensity;
(3) Networks will become more connected, have greater interaction reciprocity and a higher ratio of interactions between infected and uninfected individuals compared with solely between uninfected individuals after infection; and
(4) Changes in node, motif and network properties will be related to parasite intensity.

## 2. Methods

### (a) Ethics statement

All applicable institutional and/or national guidelines for the care and use of animals were followed. Procedures and protocols were conducted under the UK Home Office license (PPL 302 876) with approval by the Cardiff University Animal Ethics Committee.

### (b) Host–parasite system

Trinidadian guppies (*P. reticulata*) were laboratory-reared descendants of wild-caught stock from the Lower Aripo River, Trinidad, in 2012. Fish were initially housed at the University of Exeter, before transfer to Cardiff University in 2014 to be maintained in 70 l dechlorinated water tanks under standard conditions of 24 ± 0.5°C on a 12 h light: 12 h dark photoperiod (lights on 07.00−19.00). Fish were fed daily on Aquarian® Tropical fish flakes, subsidized with freshly hatched *Artemia salina* and adult *Daphnia magna*. Aquaria were checked weekly for fry, which were transferred to rearing tanks from which female fish were isolated at 8−12 weeks. Only female guppies (*n* = 120) were used due to their greater propensity to shoal than males [29], but also to avoid the confounding effects of male courtship behaviour and sexual interactions on parasite transmission and social network structure.

For experimental infections, we used the isogenic *Gt3* strain of *G. turnbulli*, which originated from a single worm isolated from an ornamental guppy in 1997. This ectoparasite population has since been maintained in culture, as described by Stewart *et al*. [30]. The monogenean worm is a common ectoparasite of guppies in both wild and ornamental stocks, and has a range of physiological and behavioural impacts [16,30,31]. It is directly transmitted, transferring from host to host when the fish contact one another and has a short generation time, giving birth to live (already pregnant) young that attach to the fish alongside the parent worm [32]. To experimentally infect a fish, an infected (donor) fish from the culture was sacrificed via cranial destruction, and the caudal fin was brought into close contact with a naive (recipient) guppy, which had been temporarily anaesthetized with 0.02% tricaine methanesulfonate (MS−222). The transfer of parasites was observed under a dissecting microscope with fibre-optic illumination, following the standard methods of King & Cable [33]. Control fish (i.e. sham infected) were handled and exposed to anaesthetic in the same manner as the experimental fish but without exposure to parasitic infection. Parasite infections were monitored non-destructively throughout the experiment by again briefly anaesthetizing each fish in a shoal (including the control shoals) and counting the number of external worms on the surface of the fish using a dissecting microscope.

### (c) Experimental set-up

Experimental trials took place in a 70 l tank of dechlorinated water, maintained under standard light and temperature conditions (see §2b). A 2 cm layer of fine gravel substrate filled the base of the aquarium, which was lit from above using daylight-mimicking strip lights (Sylvania T5 F13W/54−765 G5 Luxline Standard Daylight bulb) diffused by white fabric. The chamber was surrounded on three sides with opaque white fabric to prevent external disturbances, with one side left open to allow for observations.

### (d) Behavioural experiments

A total of 20 replicate shoals, each containing six sized-matched female *P. reticulata*, were monitored daily for 10 days, with experimental infection occurring on day 5 in 15 randomly selected replicates, and a sham infection in the remaining five controls. Each fish was uniquely marked using visual implant elastomer (VIE), enabling individual fish identification during a trial. To do this, fish were briefly anaesthetized using 0.02% MS−222, and VIE was injected into the ventral or dorsal muscle tissue. This is a marking procedure extensively used in guppies [34–37] that does not appear to influence social behaviour [38].

Fish standard length (SL; mm) was measured before each group was placed into a separate 5 l aquarium to form shoals over a 2 week familiarization period [39] before transferring to an experimental chamber to acclimate for 24 h.

On day 5, all fish were temporarily isolated in individual 1 l pots and either the most or least connected shoal member (determined by assessing accumulated contact frequency data until day 5; see §2e) was infected with exactly 30 *G*. *turnbulli* individuals. This procedure formed three experimental treatments: most connected infected (*n* = 7 shoals), least connected infected (*n* = 8 shoals) and uninfected controls (*n* = 5 shoals). The unbalanced experimental design arose through the limited availability of mature female fish for the experiment. Despite uneven sample sizes, an adequate number of replicates ensured that robust statistical analysis comparing experimental treatments could be performed. Within each infected shoal, a single fish was experimentally infected and the remaining five fish in each shoal, as well as each fish in the control groups, were sham infected by anaesthetizing and manipulating under the microscope, but without exposure to parasites. Fish were revived in 1 l of dechlorinated water and returned to their shoal groups. Infection was confirmed on day 6, and each fish was screened on consecutive days thereafter (days 7, 8, 9 and 10) to quantify *G. turnbulli* intensity, following behavioural observations. At each time step, the control and experimental groups underwent the same experimental procedures; anaesthesia followed by handling.

### (e) Social network construction

For each shoal, interactions were monitored on each day (1–10) for a 10 min period (between 9.00−12.00, three shoals per experiment). The frequency of interactions between individuals (the number of direct contact events, e.g. skin-skin contact including a bite or the brushing of fins, typically lasting <1 s) was recorded for all individuals, as well at the directionality of the interactions (i.e. which fish initiated the interaction). This resulted in a series of directed, weighted networks, where individuals are represented by nodes and interactions between individual fish by edges. Each behaviour recorded was directional such that we could record who approached who, giving us the ability to quantify the number of outgoing contacts from shoal mates (‘out degree’) and the number of incoming contacts (‘in degree’). Because multiple interactions can occur over time these edges, or interactions within the network, were weighted, i.e. were a simple count of how many times they occurred.

### (f) Data analysis

All metric calculations and statistical analyses were completed using R Statistical Software (v. 4.3.1. ‘Beagle Scouts’ [40]).

**Figure 1.**
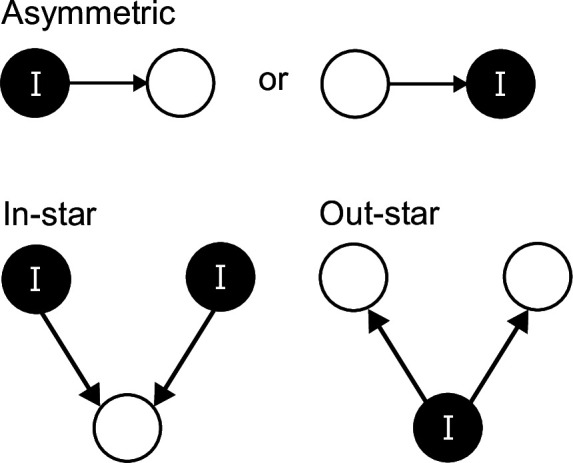
Two- and three-node motifs relevant to parasite transmission. There are other metrics involving more nodes (e.g. four or five), but as these networks were composed of six nodes in total, we restricted analysis to motifs with ≤50% of nodes.

We calculated metrics to summarize the node, motif and network-level properties of the directed, weighted networks. At the node level, weighted in- and out-degree were calculated for all individuals using the ‘strength’ function in the ‘igraph’ package [41]. We also calculated unweighted betweenness centrality using the ‘igraph’ package [41], which measures the number of times an individual lies on the shortest path between others in a network and from a disease perspective can identify individuals that may act as ‘bridges’ of transmission to otherwise unreachable individuals, or if uninfected can act as a ‘firebreak’ for infection passing through a network [42,43]. Motifs and subnetwork structures [26] were identified and enumerated across the different networks. We focused on a series of motifs that are important for parasite transmission, and provide additional information to that provided by in- and out-degree by incorporating additional interactions with shared nodes (figure 1): (i) asymmetric two-node interactions (in- and out-degree), (ii) out-star interactions from an infected fish to two other individuals (infected or uninfected) and (iii) in-star interactions between two infected individuals to another individual (infected or uninfected). For each motif type, we summarize the frequency as both count (i.e. the total number of those motifs [n]) and weighted count (i.e. the sum of the interaction strengths within those motifs [q]). As motif frequency is contingent on the number of interactions within networks, we standardized the frequencies based on total number of infected fish, i.e. converting the values into a relative frequency. We then assessed the relationships between motif frequency and the mean intensity of parasites within shoals. At the network scale, we calculated connectance, as edge density (edges/nodes^2^), to describe the degree to which the overall network is connected, as this equates to the potential for a parasite to be transmitted to all individuals if the network is fully connected. We also calculated reciprocity, the proportion of mutual connections (i.e. the probability of an opposite counterpart to a directed edge in the graph). Finally, for each network, we calculated the ratio of links from fish with higher to lower levels of infection (number of *G. turnbulli* individuals per fish), and *vice versa*, to indicate whether highly infected fish are more strongly interacting with uninfected or less infected fish.

We investigated differences in the node, motif and network properties between social networks across different treatments, before and after infection, as well as in relation to the levels of parasitism using generalized linear mixed models (GLMMs). We ran models in the ‘lme4’ package [44] and the ‘glmmTMB’ package [45] and validated model performance using the ‘DHARMa’ package [46]. Model formulae are provided in the electronic supplementary material. However, the generic model structure follows:


$$Metric∼Parasite\;intensity+Treatment^{*}Time+(1|FishID)+(1|Shoal\;ID)$$

Time was included in two ways within models: (i) before and after infection (categorical; before and after) or (ii) days (ordinal; 1−10). A mixture of Gaussian, negative binomial and Poisson model families and associated link functions were used for different metrics (see electronic supplementary material, table S1), and in some cases, zero-inflation corrections were applied.

## 3. Results

**Figure 2.**
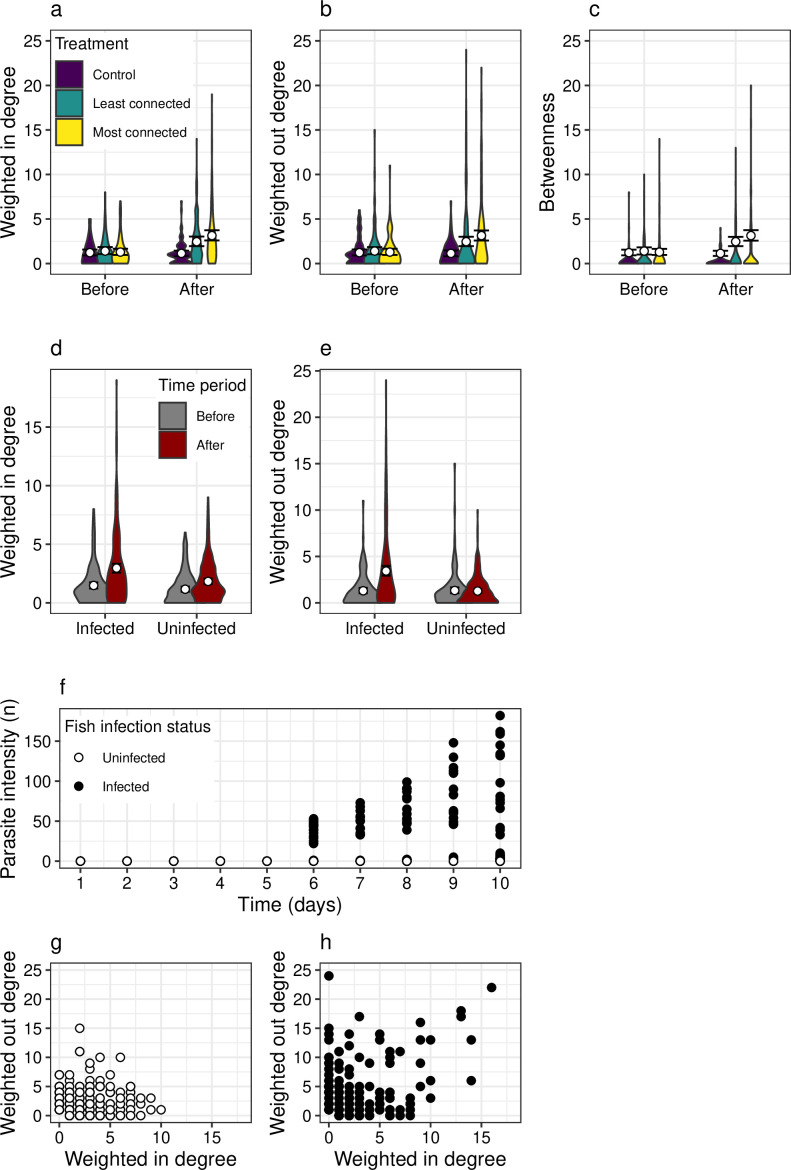
Changes in node-level metrics with parasite infection. (a–c) Weighted node metrics before and after infection across the three treatments in the experiment. (d–e) Differences in weighted degree metrics before and after infection across fish that eventually become infected or avoided infection throughout the experiment (i.e. to see whether patterns in* (*a–c) are a result of changes in the interactions for uninfected or infected fish). (*f*) Relationship between time and parasite intensity across individuals. (g–h) Relationships between weighted in- and out-degrees for uninfected and infected fish, respectively.

Over the duration of the experiments, across the experiments and in response to parasitism, node metrics varied significantly: weighted in-degree (Negative binomial GLMM: lognormal *R*^2^c = 0.26, *n* parameters = 10, *n* observations = 719, Χ^2^ = 72.28, *p* < 0.001), weighted out-degree (Zero-inflated Poisson GLMM: *R*^2^c = 0.14, *n* parameters = 11, *n* observations = 719, X^2^ = 79.83, *p* < 0.001) and betweenness (Zero-inflated Poisson GLMM: *R*^2^c = 0.26, *n* parameters = 11, *n* observations = 719, X^2^ = 84.33, *p* < 0.001). After infection with *G. turnbulli* (i.e. comparing days 1−5 against 6−10), interactions between individuals of *P. reticulata* changed (figure 2). Specifically, weighted in-degree (Wald test: X^2^ = 9.47, d.f. = 2, *p* = 0.009) increased after infection in the least and most connected treatments (figure 1a,c) but not in the controls. Weighted out-degree (Wald test: X^2^ = 78.5, d.f. = 1, *p* < 0.001) and betweenness (Wald test: X^2^ = 5.62, d.f. = 1, *p* = 0.018) both increased alongside the intensity of parasites, but did not significantly increase after infection in the least or most connected treatments in comparison to the control (Weighted out-degree Wald test: X^2^ = 2.56, d.f. = 2 p = 0.28; and betweenness Wald test: X^2^ = 0.49, d.f. = 2, *p* = 0.78). The change in node metrics was primarily due to increases in the degree values for infected fish, as opposed to those that never became infected during the study (figure 2d,e). At higher temporal resolutions (e.g. across days), as the infection trajectory progressed over time, so too did the mean in- and out-degree of fish across the shoals (figure 2g).

**Figure 3.**
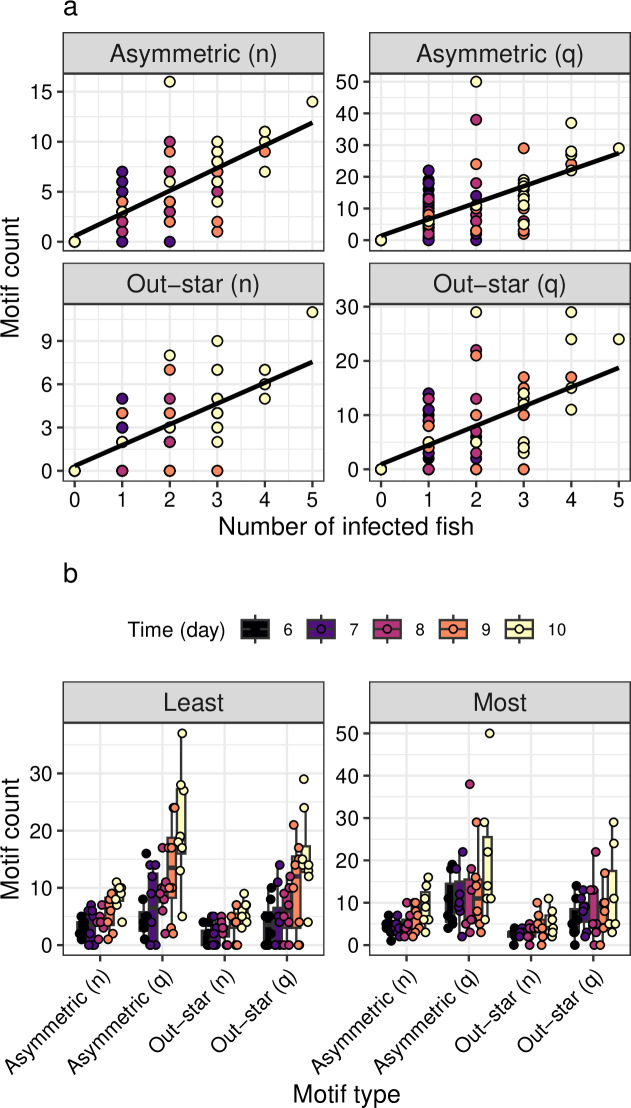
Motif frequencies post-infection (days 6−10). (a) Relationship between the number of infected fish and the frequency of motifs (best-fit lines from linear regression).(b) Relative frequency of motifs across the treatments. Motifs are either count (n) or weighted count (q) (see §2).

Motifs were variable across the treatments and time points (figure 3), but showed no significant patterns in relation to the experimental manipulations. There was not a significant increase in motifs over time after the infection (figure 3a). Although there were general increases in the relative number of motifs with mean parasite intensity in the different shoals, this was nonlinear and non-significant across the different motif types (figure 3b).

**Figure 4.**
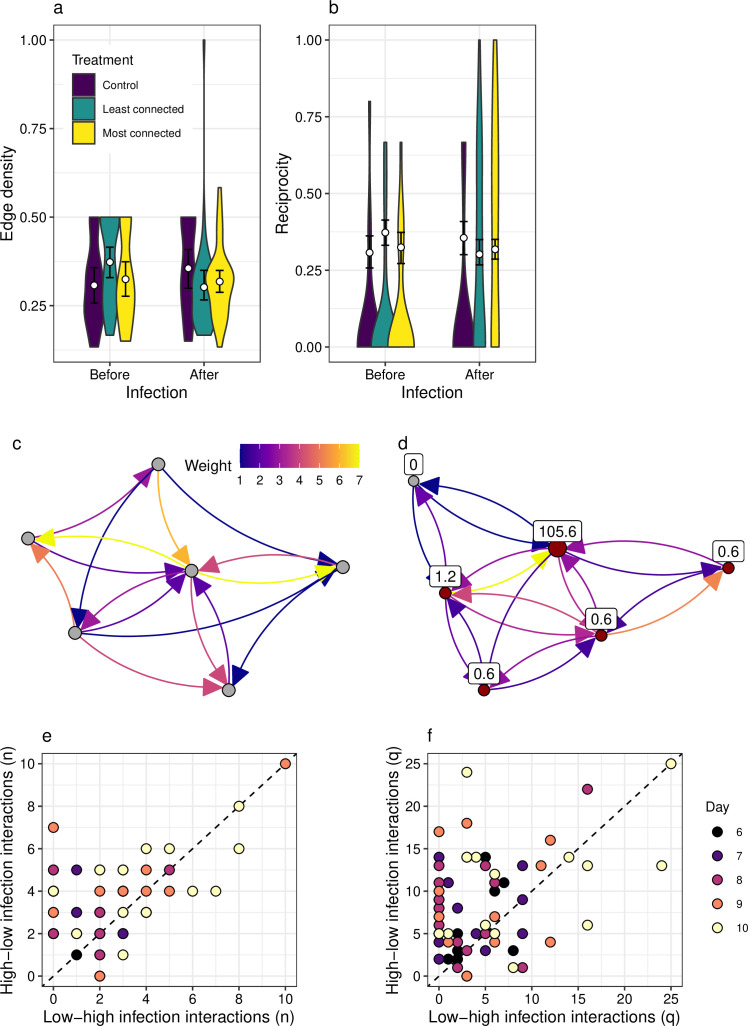
Changes in network characteristics. (a, b) Edge density (a measure of connectance) and reciprocity before and after infection across the three treatments in the experiment. (c) Aggregated network of interactions for shoal I before infection (days 1−5). (d) Aggregated network of interactions for shoal I after infection. Grey nodes indicate uninfected individuals and red nodes indicate infected individuals. Values represent mean number of parasites (days 6−10). (e) Relationships between number of interactions in the direction from low/uninfected individuals to highly infected individuals.(f) Relationships between the weight of interactions in the direction from low/uninfected individuals to highly infected individuals.

Network topology, in comparison to node and motif metrics, was far less variable across treatments (figure 4). There was no significant difference between network topological metrics (connectance/edge density and reciprocity; figure 4a,b) after infection, despite there being variation in the node characteristics and the strength of interactions varied across the pairwise interactions (e.g. figure 4c,d). With regards to the ratio of interactions from high to low infection fish, and *vice versa*, in general, across the networks, there was asymmetry—with greater frequency and weights of high to low interactions (figure 4e,f).

## 4. Discussion

Ectoparasite infection led to changes in the interactions within social networks in a shoaling fish species. Interactions between individuals switched and the frequency of these interactions changed, primarily driven by infected individuals interacting more strongly with a greater number of conspecifics. Furthermore, there was a turnover in interactions over time, with fish with greater parasite burdens interacting more strongly with fish with lower parasite burdens. Findings at the individual level provide further evidence for ‘shedding’ or ‘off-loading’ behaviour in parasitized fish. Individual-level changes in interactions, however, did not manifest themselves in significant alterations at the motif and network scale. This may indicate that the responses of social networks in fish to stressors (e.g. parasitism) are driven primarily by individual responses, as opposed to responses at the group level.

Before interpreting the results of the study further, it is important to acknowledge the following caveats of the work. Firstly, only female guppies were used during this experiment due to their greater propensity to shoal than males [47]. In the wild, male guppies move between female shoals in search of mating opportunities, which could subsequently enhance parasite transmission between individuals (see [48,49]), and thus have the potential to substantially modify social network dynamics. Secondly, the experiments in this study focused on short-term effects on behaviour, as our trials were over a limited time. Monitoring shoals until the point of parasite clearance would have provided further insight into the efficacy of the behavioural adaptations to parasitism. However, it would have also inflicted suffering on the individual fish, as we specifically started the experimental infections with a high parasite burden (to focus more on the direct host response to the parasite, rather than host immune effects) and would be in violation of the 3Rs (replacement, reduction and refinement; [50]). Thirdly, findings are relevant to directly transmitted parasites with direct life cycles. In fact, there is a significant gap in our understanding of how parasites, with indirect life cycles (i.e. those that have life stages in intermediate hosts), affect social network structure. Finally, other characteristics of the hosts (e.g. size, reproductive status, fitness, immune status; [51]) as well as the parasites are important in parasite transmission, and subsequently social network structure (based on the findings of this study). Here, we use a single fish and a single parasite species, and therefore, the wider applicability of the findings to other fish and parasites that, e.g. have different social interactions, transmission strategies or co-infections is unknown.

The linear increase in the number and strength of interactions with parasite intensity (i.e. relationship between parasite intensity and weighted-out degree), provides further support on top of the current literature for the ‘off-loading’ mechanism, as the greater the level of parasite infection the stronger the interactions and thus more intense ‘off-loading’ behaviour. ‘Off-loading’, although appearing to be an individually motivated behaviour, may actually provide a benefit at the shoal level, vaccinating conspecifics against future infections. Faria *et al*. [52], e.g. showed how guppies that had endured a primary *G. turnbulli* infection experienced significantly lower parasite intensities during secondary infections. However, it remains unclear as to whether this individual-level response has group-level benefits.

Modifications to social interactions, and the networks they form, may have knock-on effects. It can affect the fundamental ecology of an ecosystem. Intra-group interactions can drive changes in intra-specific competition and, in turn, resource utilization and consumption [53]. Changes in competition and dietary niches, in turn, could have a variety of implications for the wider structure and function of the aquatic ecosystem, e.g. the flux of energy and material through food webs (e.g. [54]). Alterations in the structure of social networks may also have implications for the resilience of individuals and populations to future biotic and abiotic changes [55]. The observed increases in the strength of interactions may have an effect on the transmission of other parasites, as has been shown in other systems (see review by [56]). Also, changes in the topology might make the network more susceptible or resilient to parasites. For example, loss of individual fish due to changes in water quantity or quality, or enhanced interspecific competition from an introduced species, may enhance parasite transmission and effects (e.g. [57,58]). Our understanding of these interactive and cascading effects of environmental change and social interactions is currently limited, yet this is a vital avenue of future research [59].

Social networks are constantly adapting to changing biotic and abiotic conditions [60]. Previous studies have shown that behaviours in fish shoals are altered by parasite infection (e.g. [61]), yet here, we identify that although the identity and strength of interactions between individuals change, there is little alteration in the overall structure of the motifs and networks. This suggests either a dynamic and adaptive response across all individuals in the social network, mitigating any changes observed at the individual level, or that other confounding factors are influencing structure. It is, therefore, important to monitor not only network structure, but also the identity, direction and strength of the individual interactions within those networks. Moving forward, it is important to understand how social networks respond to simultaneous and sequential stressors of different types (e.g. multiple stressors), while also understanding how behavioural networks affect individual and population endpoints (e.g. mortality, fecundity, growth, population dynamics). With this additional understanding, moving across levels of biological organization, it will be possible to predict the response of populations to multiple stressors, accounting for behavioural plasticity and the effects of intraspecific ecological interactions.

## Data Availability

Data and code are archived in a Zenodo repository [62]. Supplementary material is available online [63].

## References

[1] Krause J, Croft D, James R. 2003 Social networks in fish. J. Fish Biol. **63**, 235–235. (10.1111/j.1095-8649.2003.0216u.x)

[2] Redhead D, Power EA. 2022 Social hierarchies and social networks in humans. Phil. Trans. R. Soc. B **377**, 20200440. (10.1098/rstb.2020.0440) 35000451 PMC8743884

[3] Farine DR, Whitehead H. 2015 Constructing, conducting and interpreting animal social network analysis. J. Anim. Ecol. **84**, 1144–1163. (10.1111/1365-2656.12418) 26172345 PMC4973823

[4] Read JM, Eames KTD, Edmunds WJ. 2008 Dynamic social networks and the implications for the spread of infectious disease. J. R. Soc. Interface **5**, 1001–1007. (10.1098/rsif.2008.0013) 18319209 PMC2607433

[5] Pinter-Wollman N *et al*. 2014 The dynamics of animal social networks: analytical, conceptual, and theoretical advances. Behav. Ecol. **25**, 242–255. (10.1093/beheco/art047)

[6] Croft DP, James R, Ward AJW, Botham MS, Mawdsley D, Krause J. 2005 Assortative interactions and social networks in fish. Oecologia **143**, 211–219. (10.1007/s00442-004-1796-8) 15682346

[7] Sah P, Mann J, Bansal S. 2018 Disease implications of animal social network structure: a synthesis across social systems. J. Anim. Ecol. **87**, 546–558. (10.1111/1365-2656.12786) 29247466

[8] He P, Maldonado-Chaparro AA, Farine DR. 2019 The role of habitat configuration in shaping social structure: a gap in studies of animal social complexity. Behav. Ecol. Sociobiol. **73**, 9. (10.1007/s00265-018-2602-7)

[9] Seebacher F, Krause J. 2017 Physiological mechanisms underlying animal social behaviour. Phil. Trans. R. Soc. B **372**, 20160231. (10.1098/rstb.2016.0231) 28673909 PMC5498293

[10] Ezenwa VO, Archie EA, Craft ME, Hawley DM, Martin LB, Moore J, White L. 2016 Host behaviour–parasite feedback: an essential link between animal behaviour and disease ecology. Proc. R. Soc. B **283**, 20153078. (10.1098/rspb.2015.3078) PMC4843650 27053751

[11] Springer A, Kappeler PM, Nunn CL. 2017 Dynamic vs. static social networks in models of parasite transmission: predicting Cryptosporidium spread in wild lemurs. J. Anim. Ecol. **86**, 419–433. (10.1111/1365-2656.12617) 27973681

[12] Firth JA, Sheldon BC. 2015 Experimental manipulation of avian social structure reveals segregation is carried over across contexts. Proc. R. Soc. B **282**, 20142350. (10.1098/rspb.2014.2350) PMC4344146 25652839

[13] Flack JC, Girvan M, de Waal FBM, Krakauer DC. 2006 Policing stabilizes construction of social niches in primates. Nature **439**, 426–429. (10.1038/nature04326) 16437106

[14] Candolin U, Rahman T. 2023 Behavioural responses of fishes to anthropogenic disturbances: adaptive value and ecological consequences. J. Fish Biol. **103**, 773–783. (10.1111/jfb.15322) 36647916

[15] Poulin R. 1999 The functional importance of parasites in animal communities: many roles at many levels? Int. J. Parasitol. **29**, 903–914. (10.1016/s0020-7519(99)00045-4) 10480727

[16] Croft DP, Edenbrow M, Darden SK, Ramnarine IW, van Oosterhout C, Cable J. 2011 Effect of gyrodactylid ectoparasites on host behaviour and social network structure in guppies Poecilia reticulata. Behav. Ecol. Sociobiol. **65**, 2219–2227. (10.1007/s00265-011-1230-2)

[17] Barber I, Hoare D, Krause J. 2000 Effects of parasites on fish behaviour: a review and evolutionary perspective. Rev. Fish Biol. Fish. **10**, 131–165. (10.1023/A:1016658224470)

[18] Timi JT, Poulin R. 2020 Why ignoring parasites in fish ecology is a mistake. Int. J. Parasitol. **50**, 755–761. (10.1016/j.ijpara.2020.04.007) 32592807

[19] Petkova I, Abbey-Lee RN, Løvlie H. 2018 Parasite infection and host personality: Glugea-infected three-spined sticklebacks are more social. Behav. Ecol. Sociobiol. **72**, 173. (10.1007/s00265-018-2586-3) 30369708 PMC6182751

[20] Reynolds M, Arapi EA, Cable J. 2018 Parasite-mediated host behavioural modifications: Gyrodactylus turnbulli infected Trinidadian guppies increase contact rates with uninfected conspecifics. Parasitology **145**, 920–926. (10.1017/s0031182017001950) 29113619

[21] Mooring MS, Hart BL. 1992 Animal grouping for protection from parasites: selfish herd and encounter-dilution effects. Behaviour **123**, 173–193. (10.1163/156853992x00011)

[22] Møller AP, Dufval R, Allander K. 1993 Parasites and the evolution of host social behavior. In Advances in the study of behavior, pp. 65–102, vol. 22. London, UK: Academic Press. (10.1016/S0065-3454(08)60405-2)

[23] Poulin R, FitzGerald GJ. 1989 Shoaling as an anti-ectoparasite mechanism in juvenile sticklebacks (Gasterosteus spp.). Behav. Ecol. Sociobiol. **24**, 251–255. (10.1007/bf00295205)

[24] Mohammed RS, Reynolds M, James J, Williams C, Mohammed A, Ramsubhag A, van Oosterhout C, Cable J. 2016 Getting into hot water: sick guppies frequent warmer thermal conditions. Oecologia **181**, 911–917. (10.1007/s00442-016-3598-1) 26965895 PMC4912592

[25] Stephenson JF, Perkins SE, Cable J. 2018 Transmission risk predicts avoidance of infected conspecifics in Trinidadian guppies. J. Anim. Ecol. **87**, 1525–1533. (10.1111/1365-2656.12885) 30047991

[26] Milo R, Shen-Orr S, Itzkovitz S, Kashtan N, Chklovskii D, Alon U. 2002 Network motifs: simple building blocks of complex networks. Science **298**, 824–827. (10.1126/science.298.5594.824) 12399590

[27] Sosa S, Sueur C, Puga‐Gonzalez I. 2021 Network measures in animal social network analysis: their strengths, limits, interpretations and uses. Methods Ecol. Evol. **12**, 10–21. (10.1111/2041-210x.13366)

[28] Runghen R, Poulin R, Monlleó-Borrull C, Llopis-Belenguer C. 2021 Network analysis: ten years shining light on host–parasite interactions. Trends Parasitol. **37**, 445–455. (10.1016/j.pt.2021.01.005) 33558197

[29] Griffiths SW, Magurran AE. 1998 Sex and schooling behaviour in the Trinidadian guppy. Anim. Behav. **56**, 689–693. (10.1006/anbe.1998.0767) 9784218

[30] Stewart A, Jackson J, Barber I, Eizaguirre C, Paterson R, van West P, Williams C, Cable J. 2017 Hook, line and infection: a guide to culturing parasites, establishing infections and assessing immune responses in the three-spined stickleback. In Advances in parasitology (eds D Rollinson, JR Stothard), pp. 39–109, vol. **98**. London, UK: Academic Press. (10.1016/bs.apar.2017.07.001) 28942772

[31] Arapi EA, Reynolds M, Ellison AR, Cable J. 2024 Restless nights when sick: ectoparasite infections alter rest–activity cycles of diurnal fish hosts. Parasitology **151**, 251–259. (10.1017/s0031182023001324) 38372138 PMC11007282

[32] Bakke TA, Cable J, Harris PD. 2007 The biology of gyrodactylid monogeneans: the ‘Russian-doll killers’. In Advances in parasitology (eds JR Baker, R Muller, D Rollinson), pp. 161–376, vol. **64**. London, UK: Academic Press. (10.1016/S0065-308X(06)64003-7) 17499102

[33] King TA, Cable J. 2007 Experimental infections of the monogenean Gyrodactylus turnbulli indicate that it is not a strict specialist. Int. J. Parasitol. **37**, 663–672. (10.1016/j.ijpara.2006.11.015) 17224155

[34] Croft DP, James R, Thomas POR, Hathaway C, Mawdsley D, Laland KN, Krause J. 2006 Social structure and co-operative interactions in a wild population of guppies (Poecilia reticulata). Behav. Ecol. Sociobiol. **59**, 644–650. (10.1007/s00265-005-0091-y)

[35] Croft DP, Krause J, Darden SK, Ramnarine IW, Faria JJ, James R. 2009 Behavioural trait assortment in a social network: patterns and implications. Behav. Ecol. Sociobiol. **63**, 1495–1503. (10.1007/s00265-009-0802-x)

[36] Hasenjager MJ, Dugatkin LA. 2017 Familiarity affects network structure and information flow in guppy (Poecilia reticulata) shoals. Behav. Ecol. **28**, 233–242. (10.1093/beheco/arw152)

[37] Wilson ADM, Krause S, Ramnarine IW, Borner KK, Clément RJG, Kurvers RHJM, Krause J. 2015 Social networks in changing environments. Behav. Ecol. Sociobiol. **69**, 1617–1629. (10.1007/s00265-015-1973-2)

[38] Croft DP, Krause J, James R. 2004 Social networks in the guppy (Poecilia reticulata). Proc. R. Soc. B **271**, S516–9. (10.1098/rsbl.2004.0206) PMC1810091 15801620

[39] Griffiths SW, Magurran AE. 1997 Familiarity in schooling fish: how long does it take to acquire? Anim. Behav. **53**, 945–949. (10.1006/anbe.1996.0315)

[40] R Core Team. 2023 R: a language and environment for statistical computing (4.3.1) [Computer software]. R foundation for statistical computing. See https://www.R-project.org/.

[41] Csardi G, Nepusz T. 2006 The igraph software package for complex network research. InterJournal Complex Syst. **1695**, 1–9.

[42] Brandes U. 2001 A faster algorithm for betweenness centrality. J. Math. Sociol. **25**, 163–177. (10.1080/0022250x.2001.9990249)

[43] Perkins SE, Cagnacci F, Stradiotto A, Arnoldi D, Hudson PJ. 2009 Comparison of social networks derived from ecological data: implications for inferring infectious disease dynamics. J. Anim. Ecol. **78**, 1015–1022. (10.1111/j.1365-2656.2009.01557.x) 19486206

[44] Bates D, Mächler M, Bolker B, Walker S. 2015 Fitting linear mixed-effects models using lme4. J. Stat. Softw. **67**, 01 1–48. . (10.18637/jss.v067.i01)

[45] Brooks ME, Kristensen K, van Benthem KJ, Magnusson A, Berg CW, Nielsen A, Skaug HJ, Mächler M, Bolker BM. 2017 glmmTMB balances speed and flexibility among packages for zero-inflated generalized linear mixed modeling. R J. **9**, 378–400. (10.32614/RJ-2017-066)

[46] Hartig F. 2019 DHARMa: residual diagnostics for hierarchical (multi-level/mixed) regression models (0.2.3). [Computer software]. See https://cran.r-project.org/web/packages/DHARMa/vignettes/DHARMa.html.

[47] Croft DP, Arrowsmith BJ, Bielby J, Skinner K, White E, Couzin ID, Magurran AE, Ramnarine I, Krause J. 2003 Mechanisms underlying shoal composition in the Trinidadian guppy, Poecilia reticulata. Oikos **100**, 429–438. (10.1034/j.1600-0706.2003.12023.x)

[48] Richards EL, van Oosterhout C, Cable J. 2010 Sex-specific differences in shoaling affect parasite transmission in guppies. PLoS One **5**, e13285. (10.1371/journal.pone.0013285) 20949014 PMC2952601

[49] Richards EL, van Oosterhout C, Cable J. 2012 Interactions between male guppies facilitate the transmission of the monogenean ectoparasite Gyrodactylus turnbulli. Exp. Parasitol. **132**, 483–486. (10.1016/j.exppara.2012.09.019) 23047132

[50] Sneddon LU, Halsey LG, Bury NR. 2017 Considering aspects of the 3Rs principles within experimental animal biology. J. Exp. Biol. **220**, 3007–3016. (10.1242/jeb.147058) 28855318

[51] Jog MG, Sackett ME, Kisty SD, Hansen JA, Stephenson JF. 2022 The behaviour of infected guppies depends on social context, parasite tolerance and host sex. Anim. Behav. **187**, 97–104. (10.1016/j.anbehav.2022.03.001)

[52] Faria PJ, van Oosterhout C, Cable J. 2010 Optimal release strategies for captive-bred animals in reintroduction programs: experimental infections using the guppy as a model organism. Biol. Conserv. **143**, 35–41. (10.1016/j.biocon.2009.06.002)

[53] Sheppard CE, Heaphy R, Cant MA, Marshall HH. 2021 Individual foraging specialization in group-living species. Anim. Behav. **182**, 285–294. (10.1016/j.anbehav.2021.10.011)

[54] Bossier S, Nielsen JR, Neuenfeldt S. 2020 Exploring trophic interactions and cascades in the Baltic Sea using a complex end-to-end ecosystem model with extensive food web integration. Ecol. Model. **436**, 109281. (10.1016/j.ecolmodel.2020.109281)

[55] Villegas-Ríos D, Jacoby DMP, Mourier J. 2022 Social networks and the conservation of fish. Commun. Biol. **5**. (10.1038/s42003-022-03138-w) PMC8885690 35228664

[56] White LA, Forester JD, Craft ME. 2017 Using contact networks to explore mechanisms of parasite transmission in wildlife. Biol. Rev. **92**, 389–409. (10.1111/brv.12236) 26613547

[57] Lafferty KD, Kuris AM. 1999 How environmental stress affects the impacts of parasites. Limnol. Oceanogr. **44**, 925–931. (10.4319/lo.1999.44.3_part_2.0925)

[58] Poulin R, Paterson RA, Townsend CR, Tompkins DM, Kelly DW. 2011 Biological invasions and the dynamics of endemic diseases in freshwater ecosystems. Freshw. Biol. **56**, 676–688. (10.1111/j.1365-2427.2010.02425.x)

[59] Blumstein DT, Hayes LD, Pinter-Wollman N. 2023 Social consequences of rapid environmental change. Trends Ecol. Evol. **38**, 337–345. (10.1016/j.tree.2022.11.005) 36473809

[60] Fisher DN, Kilgour RJ, Siracusa ER, Foote JR, Hobson EA, Montiglio PO, Saltz JB, Wey TW, Wice EW. 2021 Anticipated effects of abiotic environmental change on intraspecific social interactions. Biol. Rev. **96**, 2661–2693. (10.1111/brv.12772) 34212487

[61] Demandt N, Praetz M, Kurvers RHJM, Krause J, Kurtz J, Scharsack JP. 2020 Parasite infection disrupts escape behaviours in fish shoals. Proc. R. Soc. B **287**, 20201158. (10.1098/rspb.2020.1158) PMC7735259 33143588

[62] Reynolds M, Windsor F, Cable J, Perkins S. 2025 Data for: Parasites alter interaction patterns in fish social networks. Zenodo (10.5281/zenodo.15275988) PMC12115853 40425167

[63] Reynolds M, Windsor F, Perkins S, Cable J. 2025 Supplementary material from: Parasites alter interaction patterns in fish social networks. Figshare (10.6084/m9.figshare.c.7829406) PMC12115853 40425167

